# The shared genetic risk factors between Tourette syndrome and obsessive-compulsive disorder

**DOI:** 10.3389/fneur.2023.1283572

**Published:** 2023-10-13

**Authors:** Mohamed Adil Shah Khoodoruth, Foysal Ahammad, Yasser Saeed Khan, Farhan Mohammad

**Affiliations:** ^1^College of Health and Life Sciences, Hamad Bin Khalifa University, Doha, Qatar; ^2^Child and Adolescent Mental Health Service, Hamad Medical Corporation, Doha, Qatar

**Keywords:** Tourette sydrome, obsessive – compulsive disorder, shared genetic factors, neurogenetics, neuropsychiatic genetics, genetics, epigenetics, genomics

## Abstract

Tourette syndrome (TS) and obsessive-compulsive disorder (OCD) are two neuropsychiatric disorders that frequently co-occur. Previous evidence suggests a shared genetic diathesis underlying the comorbidity of TS and OCD. This review aims to comprehensively summarize the current literature on the genetic factors linked with TS and its comorbidities, with a focus on OCD. Family studies, linkage analysis, cytogenetic studies, and genome-wide association studies (GWAS) have played a pivotal role in identifying common and rare genetic variants connected with TS and OCD. Although the genetic framework of TS and OCD is complex and multifactorial, several susceptibility loci and candidate genes have been identified that might play a crucial role in the pathogenesis of both disorders. Additionally, post-infectious environmental elements have also been proposed to contribute to the development of TS-OCD, although the dynamics between genetic and environmental factors is not yet fully understood. International collaborations and studies with well-defined phenotypes will be crucial in the future to further elucidate the genetic basis of TS and OCD and to develop targeted therapeutic strategies for individuals suffering from these debilitating conditions.

## Graphical Abstract

Genetic studies indicate a complex interplay of multiple genes contributing to TS and OCD, with a potential shared genetic diathesis in certain families leading to the manifestation of either or both disorders. GWAS have identified significant genetic overlap between TS and OCD, including SNPs in the LINC01122 gene, suggesting a common genetic nexus for both conditions. Environmental factors such as post-infection autoimmune reactions (GABHS) and psychosocial stressors play a role in exacerbating TS and OCD symptoms, highlighting the need for a holistic approach to understanding these disorders. The evolving field of pharmacogenomics in TS and OCD treatment was discussed, with emphasis placed on the importance of personalized pharmacological interventions tailored to individual genetic variations, particularly in drug metabolism pathways. The emerging role of epigenetic modulation in TS and OCD comorbidity was explored, with emphasis placed on the need for comprehensive research into epigenetic mechanisms and their potential impact on diagnosis and treatment approaches in the future. GABHS, Group a beta-hemolytic streptococci; GWAS, Genome-wide associated studies; LINC01122, Long intergenic non-protein coding RNA 1122; SNP, Single nucleotide polymorphism; OCD, obsessive-compulsive disorder; TS, Tourette syndrome.

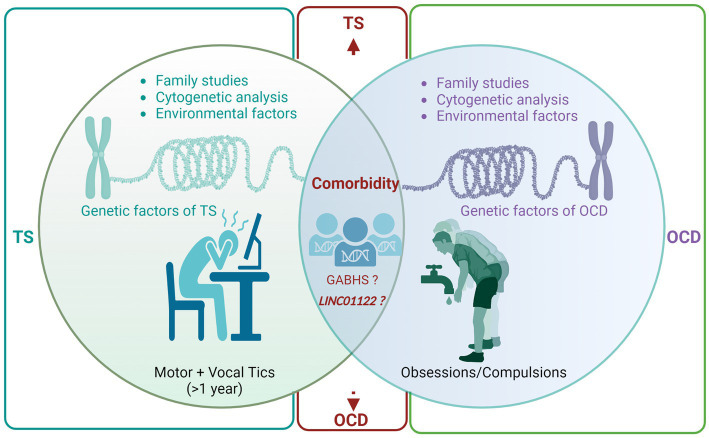

## Introduction

More than a hundred years ago, the pioneering French neurologist, Gilles de la Tourette, delineated Tourette syndrome (TS) in nine individuals exhibiting repetitive motor and vocal tics during their childhood (1). For a considerable period, TS was erroneously deemed psychogenic until the late 1960s when Drs. Arthur and Elaine Shapiro reignited scientific curiosity in the disorder by proposing its organic nature (1). Subsequent research has tremendously enhanced our comprehension of TS, categorizing it as a multifaceted neurodevelopmental and neuropsychiatric disorder. TS is estimated to afflict 0.1 to 1% of the general population and 1% of school-going children, exhibiting a conspicuous male bias, being 2–10 times more prevalent in males than females (2, 3). A significant proportion of TS patients concurrently suffer from other psychopathologies, notably obsessive-compulsive disorder (OCD), marked by obsessions and/or compulsions that substantially hinder daily functioning (4). Intriguingly, Gilles de la Tourette’s initial characterization of TS noted the presence of obsessions and compulsions among the affected individuals, thereby implying a shared genetic predisposition (1).

Tourette syndrome (TS), attention deficit hyperactivity disorder (ADHD), autism spectrum disorder (ASD), and OCD are intricate neurodevelopmental disorders with a myriad of common and rare genetic factors influencing their onset (5–9). Although recent studies have pinpointed 12 significant genetic loci for ADHD (10) and five for ASD (11, 12), only a single significant genetic locus has been recently discerned for TS (13), and, strikingly, none for OCD (14).

This concise review endeavors to elucidate the prevailing understanding of the most prominent concepts associated with the genetic underpinnings of TS and OCD. Moreover, the review accentuates the conceivable influence of environmental risk factors on the manifestation of these neuropsychiatric conditions. Lastly, the review contemplates the directions for forthcoming research on TS and OCD genetics, encompassing the potential identification of novel therapeutic targets. Ultimately, a more profound comprehension of the complex interplay between genetic and environmental determinants precipitating the onset of these disorders is imperative for devising more efficacious interventions and enhancing the prognosis for affected individuals.

### Heritability and familial clustering of Tourette syndrome: evidence from family-based studies

Robust evidence underscores the heritability of TS, a neurodevelopmental condition. Family studies have invariably demonstrated the familial aggregation of TS and associated tic disorders, evidenced by a higher prevalence of the disorder among immediate relatives relative to the general population (15). For instance, the prevalence of TS is approximately 5% in sisters and exceeds 10% in brothers, which signifies a ten-to thirty-fold elevated risk compared to the general population, considering that the estimated prevalence estimate of TS in child population varies between 0.3 and 0.9% (16). Additionally, gender-specific phenotypes have been recognized, with females exhibiting a propensity toward obsessive-compulsive disorders or behaviors, whereas males predominantly manifest TS and tics. Furthermore, genomic imprinting appears to modulate the phenotypic expression of TS, as illustrated by a study revealing a parent-of-origin effect, whereby an earlier onset is observed in offspring inheriting the disorder from their mother (17, 18).

While the familial distribution of TS suggests a genetic association, other determinants, such as infectious or environmental risk factors, might also be contributory (19). Twin studies have yielded unambiguous evidence of the proportional contributions of genetics and the environment to TS, revealing a notably increased concordance in monozygotic twins in comparison to dizygotic twins. Concordance rates among monozygotic twins have been reported to range from 53 to 56% and even reach 100% when the study methodology encompassed firsthand patient assessment and the diagnosis of chronic tics in conjunction with TS (20–22).

Numerous investigations have endeavored to elucidate the mode of TS’s inheritance, with segregation analyses proposing its transmission as an autosomal dominant trait with incomplete penetrance (23–25). Conversely, other studies have posited that TS may emanate from a major effect gene, with supplementary contributions from the genetic background and environmental determinants (26). However, a coherent depiction of the precise mode of inheritance remains elusive (27). TS likely originates from the interaction of multiple gene alleles, each exerting relatively modest effects compared to Mendelian disorders, and epigenetic influences culminating in diverse phenotypic outcomes.

Interestingly, the paradox of pronounced heritability without identified definitive mutations is not unique to TS. Essential Tremor (ET) is another classical example where, despite clear heritability and extensive research, the responsible mutations remain elusive (28). Several candidate genes have been implicated in ET, much like in TS, but a conclusive link has yet to be established (28). This phenomenon is not uncommon, as only a fraction of familial diseases, such as hereditary ataxias or dementias, have confirmed mutations (29, 30). The acknowledgment of this paradox enriches our understanding of the complexities involved in genetic research and the heritability of certain disorders.

### Interplay of genetic factors in Tourette syndrome and associated psychiatric disorders

In clinical and population-based samples, the occurrence of TS is more frequently associated with comorbidities rather than as an isolated condition, illustrating the concept that comorbidity is the ‘exception rather than the rule’. A notable comorbidity is ADHD, a neurodevelopmental disorder characterized by a persistent pattern of inattention and/or hyperactivity-impulsivity that impairs functioning or development, which has a prevalence of 54.3% among individuals with TS (4, 31). This concurrent manifestation significantly contributes to disruptive behaviors and academic challenges (32). The potential shared genetic etiology between these two neurodevelopmental disorders has been a subject of debate (33). Although there is evidence supporting the independent transmission of these disorders within families, the high comorbidity rate suggests the possibility of a shared genetic component, at least in certain TS-ADHD subgroups. A latent class analysis involving 952 individuals from 222 TS families indicated that those with TS, ADHD, and OCD might have the most heritable form of the disorder (34). Moreover, seven genes, namely *DRD2, HRH3, MAOB, BDNF, SNAP25, and SLC6A4*, have been identified in the pathogenesis of both TS and ADHD, hinting at a shared pathogenic pathway (35).

Additionally, there is a higher incidence of co-occurrence of TS and ASD compared to the general population (36). A study into genomic copy number variations (CNVs) in TS showed a notable overlap between rare CNVs associated with TS and ASD, albeit no such overlay was observed with schizophrenia or intellectual disability (37). Additionally, these neurodevelopmental disorders share genetic underpinnings involving genes such as *CNTNAP2* and *IMMP2L*, as well as the neurexin gene family (38).

The coexistence of depressive symptoms with TS may be attributed to either a shared genetic predisposition or the psychological ramifications of living with tics. A Taiwanese study involving 1,337 TS patients revealed a significantly higher incidence of depression (4.85 times more) among TS patients compared to those without TS (39). The underlying cause of this shared genetic association remains unclear. Some researchers postulate that depression is integral to TS and that both disorders share a similar genetic foundation (40). Conversely, other experts contend that TS and depression are genetically distinct, and depression may arise from the emotional struggles associated with managing a chronic illness (41). Table 1 provides an overview of studies exploring the genomics of comorbid TS and other mental health disorders.

**Table 1:** Shows studies highlighting the genomics of comorbid Tourette syndrome with other psychiatric disorders.

Study	Potential candidate genes and SNPs	Sample size	Population	Pathway/pathology
TS-ADHD				
Gunther et al. (35)	*BDNF, DRD2, HRH3, MAOB, SLC6A4, SNAP25*	26 subjects with TS, who were not on any medication, aged between 7 and 13 years old	Not reported	Catecholamine-and basal ganglia-related pathways
Abelson et al. (42)	*SLITRK1* (rs193302861 variant)	174 unrelated probands	93% Caucasian	Neurite outgrowth. Regulation occurs developmentally in the corticostriatal-thalamocortical circuits of the mouse, monkey, and human brain, initiating during embryogenesis
TS-ASD				
Fernandez et al. (37)	*FHIT, CNTNAP2, ASTN2*	460 TS cases vs. 1,131 controls	European	*FHIT*: Regulation of DNA replication and signaling stress responses. *CNTNAP2*: Development and function of neuronal circuitry and language development. *ASTN2*: Neuronal development and migration
Carias and Wevrick (38)	*CACNA1G, GRIK5, NOTCH1, PCM1, RP1L1, RPGRIP1L, ACHE, DDR2, ITGB1*	119 autistic children with TS vs. 2,603 autistic children	Simons simplex collection	Regulation of exocytosis dependent on calcium ions, transition zone of cilia, and binding of collagen
*TS-OCD*				
Verkerk et al. (43)	*CNTNAP2*	A family with TS: father and two children were affected	Not reported	Altered distribution of potassium channels within the nervous system
Abelson et al. (42)	*SLITRK1* (rs191284403 variant, also termed var321)	174 unrelated TS probands	93% Caucasian	Neurite outgrowth. Regulated during development in the corticostriatal-thalamocortical circuits of mouse, monkey, and human brains, beginning in embryogenesis
Ercan-Sencicek et al. (44)	*HDC*	A non-consanguineous family spanning two generations with nine members affected by TS, of which four also had OCD	Not reported	The biogenic amine, histamine, plays a crucial role in modulating neurotransmission. The conversion of histidine to histamine is catalyzed by the enzyme L-histidine decarboxylase
Song et al. (45)	*SLITRK5*	377 subjects with OCD vs. 1,092 controls	European American (68%) and Hispanic chromosomes (11%)	*SLITRK5* belongs to the SLITRK family, a group of proteins mainly expressed in neural tissues, possessing the ability to modulate neurite activity
Cappi et al. (46)	*CHD8, SCUBE1*	A comparison between 222 parent–child trios with OCD and 777 healthy trios	Trios were recruited at three sites: Brazil, Canada, and United States	*CHD8*: A chromatin-remodeling factor that is ATP-dependent and predominantly expressed in the developing brain. It regulates the transcription of beta-catenin (CTNNB1) target genes. *SCUBE1*: Involved in platelet activation and adhesion processes
Yang et al. (4)	*CADM2, LY6G6F, MEGT1, APOM,* and *LINC01122*	93,294 individuals, 6,788,510 markers (meta-analysis)	Not reported	*CADM2:* Extracellular recognition and intercellular adhesion. *LY6G6F*: immune system and cellular recognition. *MEGT1*: signal transduction *APOM*: lymphocyte trafficking

Studies are listed in chronological order. *ACHE,* Acetylcholinesterase*; APOM*, Apolipoprotein M; *BDNF*, Brain-derived neurotrophic factor; *CACNA1G,* Calcium channel, voltage-dependent, t type, alpha-1 g subunit; *CADM2*, Cell adhesion molecule 2; *CHD8,* Chromodomain helicase DNA-binding protein 8; *CNTNAP2*, Contactin-associated protein 2 gene; *CTNNB1*, Cadherin-associated protein, beta; *DRD2*, Dopamine Receptor D2; *FHIT*, fragile histidine triad; *GRIK5,* Glutamate receptor, ionotropic, kainate 5; *HDC*, Histidine decarbocylase; *HRH3*, Histamine receptor H3; *ITGB1*, Integrin, beta-1; *LINC01122*, long intergenic non-protein coding RNA 1122; *LY6G6F*, Lymphocyte antigen 6 family, member G6F; *MAOB*, Monoamine oxidase B; *MEGT1*: Megakaryocyte-enhanced gene transcript 1; *NOTCH1,* Notch receptor 1; OCD, obsessive-compulsive disorder; *PCM1,* Pericentriolar material 1; *RPGRIP1L,* RPGRIP1-like; *RP1L1,* RP1-like protein 1; SCUBE1, Signal peptide-, cub domain-, and EGF-like domains-containing protein 1; *SLC6A4*, Solute carrier family 6 (neurotransmitter transporter, serotonin), member 4; *SLITRK1*, Slit and Trk-like 1; SLITRK5, SLIT and NTRK-Like Family, Member 5; SNAP25, Synaptosomal-associated protein, 25 kDa; SNP, single nucleotide polymorphisms.

## Decoding the genetic nexus between TS and OCD

### Family studies

Studies conducted globally have estimated that OCD affects 2–3% of the population throughout their lifetime (47). family studies have revealed that 30 to 60% of probands with TS also meet the diagnostic criteria for OCD (23, 48), signifying an elevated risk of OCD in family members of individuals with TS. However, this elevated risk does not necessarily infer a singular genetic predisposition leading to different manifestations. It could be attributed to common pathophysiological mechanisms or aggregation of environmental hazards rather than a shared genetic background. Nonetheless, several studies reported higher OCD rates (without tics) among TS patients’ relatives who do not exhibit obsessive-compulsive symptoms compared to unaffected families (21, 23, 48–50). Conversely, family studies involving OCD probands consistently revealed increased tic disorder rates (50–52). Moreover, multiple clinical case series indicated that individuals with tic-related OCD frequently exhibit symmetry and precision obsessions, a need to repeat activities for completion, or a ‘just right’ sensation (53, 54). In summary, the high OCD prevalence in TS patients and their families, obsessive-compulsive symptom patterns in TS pedigrees, and a distinct natural history of tic-related OCD indicate that a shared genetic diathesis in some families could manifest as TS, OCD, or both.

### Linkage analysis

The implication of genetics in disorders was elucidated through research on families and twins, which necessitated identifying specific genetic loci involved. The autosomal dominant inheritance pattern observed in studies marked TS as a prime candidate for linkage analysis. Yet, despite years of parametric linkage studies, no specific genetic locus, except for the HDC gene, which codes for the enzyme L-histidine decarboxylase, has been pinpointed in the etiology of this neurodevelopmental disorder. L-histidine decarboxylase is the rate-limiting enzyme in histamine biosynthesis (55). A rare functional mutation in the HDC gene resulting in loss of enzyme activity was identified by Ercan-Sencicek et al. in a nonconsanguineous two-generation family with nine affected members, four of whom had OCD (44). Additionally, Fernandez et al. found a robust association between rare copy variants in families with TS and histaminergic signaling, notably in the H1 receptor pathways (37). These significant findings implicate that histaminergic neurotransmission could be involved in TS’s pathobiology, not only in this particular family but possibly on a broader scale.

Histamine (HA) signaling within the central nervous system is mediated by four 7-transmembrane G protein-coupled receptors, both pre-synaptically (predominantly H3 and H4) and post-synaptically (H1–H3), with presynaptic HA receptors being responsible for regulating the release of HA and dopamine (56). Existing evidence suggests a counter-regulatory role of HA, wherein decreased HA results in increased dopamine signaling and vice versa (57). The H2 and H3 receptors, enriched in the striatum and cortex, are critical brain regions implicated in TS (55). In TS, an excess of dopamine in the striatum is believed to stimulate the thalamocortical circuits, a theory substantiated by the effectiveness of dopamine blockers in suppressing TS symptoms (58). Recently, activating the histaminergic afferent system, particularly H3, has ameliorated obsessive-compulsive-like behaviors (59). These findings hold promise for developing innovative therapeutic strategies for TS and OCD.

### Consanguinity

Investigating the potential contribution of recessive genetic variations to reduced penetrance of TS is a complex task. However, autozygosity, which occurs when both alleles at a genetic location are inherited from a single ancestor, allows the application of homozygosity mapping even in small families. This technique has been used to detect recessive variations in highly consanguineous populations (60). Motlagh et al. identified 12 Iranian families with TS characterized by early emergence of vocal tics, coprolalia, and, often, coexisting OCD (61). Analyzing the genetic constitution of affected and unaffected family members could uncover rare recessive variations associated with the comorbidity of TS and OCD.

### Cytogenetic studies

Cytogenetic techniques such as karyotyping, fluorescent *in situ* hybridization, and array comparative genomic hybridization are valuable tools for detecting chromosomal irregularities like translocations, deletions, and duplications. By identifying genes located near these disrupted regions, potential genes that may increase susceptibility can be identified for further study. Notable chromosomal abnormalities linked with TS familes have been found on several chromosomes, including 2p12, 3p21.3, 7q35-36, 8p21.4, partial trisomy 9, 13q31, and 18q22.3 (43, 62–64).

State and colleagues described a patient with chronic tics (CT) and OCD, who displayed a specific inversion on chromosome 18q22 (62). The telomeric end of this inversion was close (within 1 Mb) to a translocation breakpoint earlier noted by another pedigree study (65). Although the inversion did not structurally alter any genes, functional analysis of two transcripts in that area showed changes in replication timing. This hints at the influence of epigenetics on gene activity, indicating that genes in this zone might be relevant for TS.

Cuker et al. described a translocation t (2, 18)(p12;q22) in a 14-year-old female exhibiting pronounced symptoms of OCD and CT (64). Interestingly, this patient’s breakpoint on chromosome 18 was in proximity (approximately 5 Mb) to two previously recognized rearrangements linked to TS, OCD, and CT, suggesting that this particular chromosomal region may house pivotal genes associated with the TS/OCD symptom range (62, 65).

In another case, Ververk and colleagues studied a family with a history of TS and OCD, identifying a complicated chromosomal anomaly that affected the *CNTNAP2* gene, a massive gene in our genome (43). This gene, which comprises 25 exons and spans 2.0 Mb, codes for a membrane protein situated at a distinct point at the nodes of Ranvier of axons (66). They proposed that *CNTNAP2* could be a key gene for TS/OCD due to its role in signal transmission, a process known to be disrupted in this combined disorder (43).

### Genome-wide associated studies (GWAS)

The initial genome-wide examination of large and rare CNVs in OCD has identified a recurrent deletion in the 16p13.11 genomic region (67). This specific deletion appeared in individuals with both OCD and TS and those with only OCD, suggesting a potential genetic association with both conditions. Furthermore, the study unveiled genomic alterations in the 22q11 region in several participants with TS and OCD, corroborating findings from an earlier report on velocardiofacial syndrome (68).

A recent meta-analysis investigated the genetic overlap between TS and OCD using GWAS data (4). This research identified 21 significant genome-wide SNPs located in the *LINC01122* gene on chromosome 2p16.1, all manifesting a consistent directional impact on both conditions. Notably, this specific region was absent in prior significant findings from a comprehensive analysis spanning eight psychiatric disorders, implying its potential uniqueness to the TS-OCD linkage (69). These results allude to a substantial genetic convergence between TS and OCD, potentially guiding the formulation of innovative therapeutic approaches. Concurrently, the study brought to light four novel genes—*CADM2, LY6G6F, MEGT1,* and *APOM*—previously associated with diverse neurological activities. Nevertheless, subsequent in-depth functional evaluations are necessary to clarify the exact functions of these genes in predisposing individuals to TS and OCD, as well as their viability as treatment targets.

**Figure 1:**
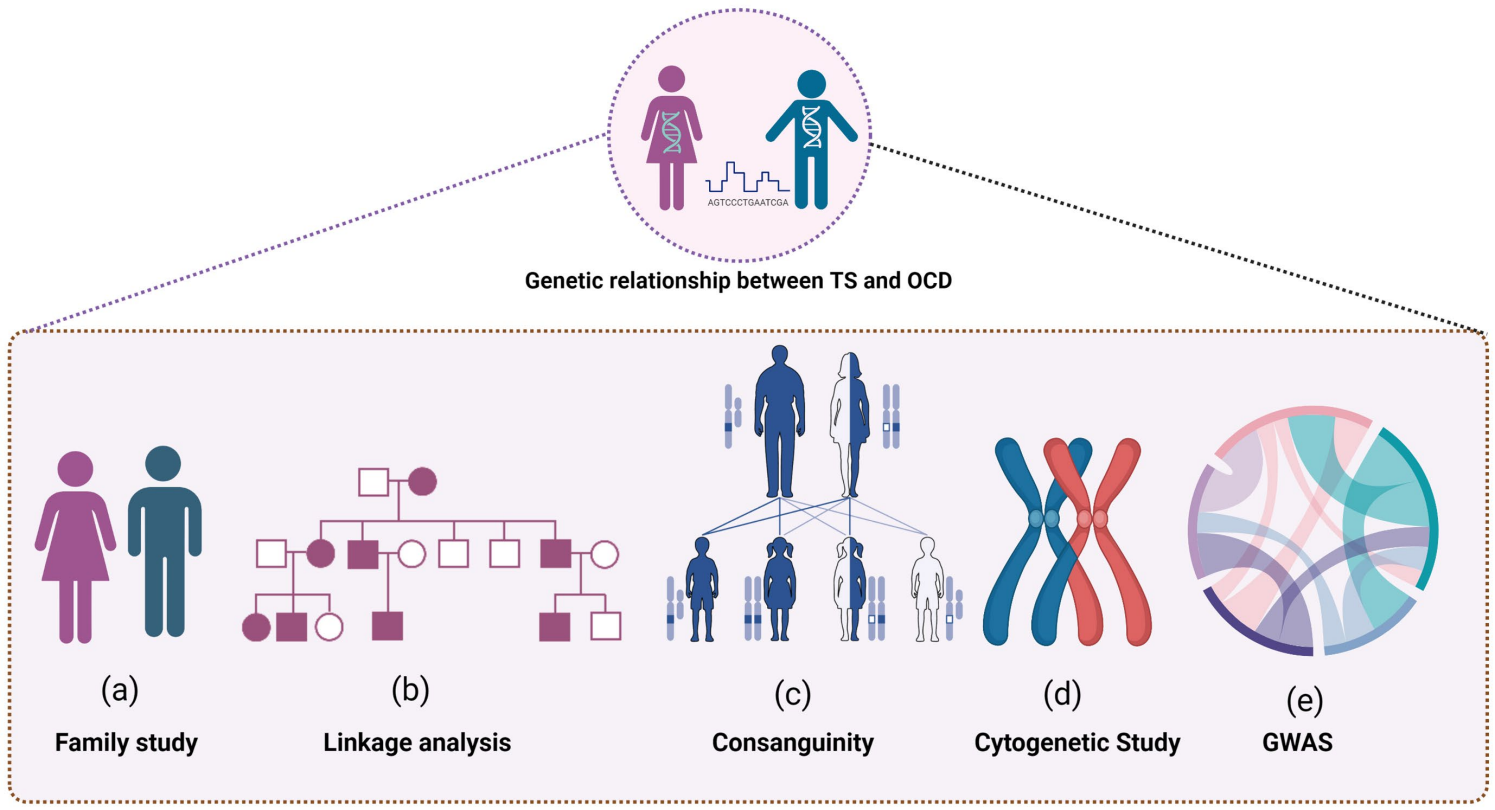
Several research methodologies were employed to uncover the intricate connections, including family studies to explore the hereditary nature of TS and OCD, and linkage analysis to pinpoint the specific genetic loci involved. The role of consanguinity was also examined, shedding light on how shared genetic backgrounds could influence the prevalence of TS and OCD. Cytogenetic studies offered insights into chromosomal variations and abnormalities associated with the conditions, contributing to the broader understanding of their genetic underpinnings. Furthermore, the figure highlights the findings from Genome-Wide Association Studies (GWAS), which helped to identify potential genetic markers and risk alleles associated with the development of both disorders.

Figure 1 provides a detailed visualization of the extensive research conducted to understand the genetic relationship between TS and OCD.

## Environmental factors

Multiple environmental contributors have been proposed to influence the onset of TS, including complications during pregnancy and delivery, androgen exposure, thermal stress and fatigue, post-infection autoimmune reactions, and psychosocial distress. This review will focus specifically on the last two.

The proposition that post-infectious autoimmune responses might cause TS and OCD originated in the 1800s (70) and remains a contentious subject in contemporary research (71). It is recognized that Group A beta-hemolytic streptococci (GABHS) can spark immune-driven conditions in genetically susceptible individuals. In 1998, Swedo and colleagues documented a cohort of patients manifesting OCD and TS symptoms concurrent with GABHS exposure (19). They postulated that GABHS-triggered immune reactions target the basal ganglia, a neural region previously implicated in other movement disorders. Consequently, they introduced the concept that Pediatric Autoimmune Neuropsychiatric Disorder Associated with Streptococcal infection (PANDAS) could be a discrete clinical category encompassing certain TS and OCD cases.

Moreover, TS patients frequently report elevated psychosocial stress, and latent class modeling on longitudinal datasets indicated that preceding stress events might amplify the intensity of obsessive-compulsive manifestations (72). Interestingly, Lin et al. found that GABHS infections could modestly predict future exacerbations in tic and OCD symptom intensity and further strengthen the prognostic value of existing psychosocial stress on ensuing tic and OCD symptom intensification (73). Buse et al. proposed a hypothesis suggesting stress might modulate tic pathology via dopaminergic and noradrenergic signaling pathways and immune mechanisms (74). They also indicated the possibility of mutual interactions between psychosocial stress and TS-associated aberrations in neurotransmission or immune responses. A study by Adams et al. demonstrated that stress can worsen OCD symptoms, impacting neural pathways like the corticostriatal and limbic systems, which results in neuronal growth in specific regions and shrinkage in others (75). Such neural shifts may foster an imbalance in purposeful versus routine/habitual behaviors, which could underlie the development and manifestation of OCD characteristics. Collectively, these insights underline the intricate relationships between GABHS infections, psychosocial stressors, and the severity of TS and OCD symptoms, highlighting opportunities for innovative therapeutic strategies.

## Future directions and conclusion

### Epigenetic dimensions

The domain of epigenetic modulation is rapidly emerging as a potentially pivotal area for decoding the intertwined etiologies of TS and OCD comorbidity. Existing evidence compellingly delineates specific ‘developmental windows’ wherein the genetically anchored micro-circuitry of crucial limbic-hypothalamic-midbrain architectures is vulnerable to early environmental factors. These early exposures appear to profoundly dictate an individual’s reactivity to psychosocial challenges and their aptitude for nurturing subsequent generations. Investigations have shown that these initial environmental interactions can alter methylation patterns within the promoter region of the glucocorticoid receptor gene, influencing the hypothalamic–pituitary–adrenal (HPA) stress response. Notably, pronounced maternal stress during gestation has been linked to a more complex form of TS (76). In tandem, cross-sectional and longitudinal research concerning TS and early-onset OCD imply a heightened sensitivity of these conditions to psychosocial stress (72). TS-affected individuals have been observed to exhibit an augmented stress reaction mediated by the HPA pathway (77, 78), accompanied by increased cerebrospinal fluid (CSF) concentrations of corticotropin-releasing hormone (79). Such observations resonate with epigenetic manifestations, as described by Meaney et al. (80).

### Pharmacogenomic perspectives

Today, physicians are moving away from a “one size fits all” model to one that considers individual patient differences. The ultimate goal of pharmacogenomics is to maximize efficacy and minimize adverse effects. Practice parameters have suggested a difference in pharmacological interventions between tic-associated and non-tic-associated OCD. For example, while selective serotonin reuptake inhibitors (SSRIs), metabolized by the CYP2D6 and CYP2C19 enzymes, are efficacious for treating OCD, their response appears to be less favorable for tic-associated OCD (81, 82). Conversely, alpha-2a agonists are proficient in addressing tics but not OCD (83). A meta-analysis found that there is significant inter-ethnic variation in the prevalence of phenotypes predicted by CYP2D6 and CYP2C19, which are clinically relevant for pharmacological treatment, and thus pre-emptive pharmacogenetic testing should be considered for patients undergoing therapy with drugs metabolized by these pathways (84). Following this, The U.S. Food & Drug Administration (FDA) then recommended therapeutic recommendations for some pharmacogenetic associations of SSRIs, such as citalopram, escitalopram, and fluvoxamine, as well as clomipramine, considering the patient’s metabolizing status (85). Therefore, pharmacogenomic-based prescribing remains an exciting field to empower psychiatrists and neurologists to deliver better, more personalized care to patients with comorbid TS and OCD.

## Conclusion

Research spanning over the past 40 years has shown that the genetic landscape of TS is considerably more diverse and intricate than previously perceived. Significant strides in discerning the genetic underpinnings of TS and its concurrent disorders like OCD have been achieved by leveraging family-based studies, segregation analyses, linkage analysis, GWAS, and investigations into rare genetic variants. Existing data indicates a genetic overlap between TS and OCD. The susceptibility to TS-OCD may be shaped by the cumulative impact of several genes interplaying with distinct environmental determinants, such as psychosocial and infectious stressors. Larger international genetic, epigenetic, and pharmacogenomic studies with well-defined phenotypes in the current era of novel genetic discoveries, will have tremendous ramifications for the tailored diagnosis and therapeutic approaches to comorbid TS and OCD.

## Author contributions

MK: Writing – original draft, Writing – review & editing, Conceptualization, Methodology, Funding acquisition. FA: Visualization, Writing – review & editing. YK: Writing – review & editing. FM: Funding acquisition, Resources, Supervision, Writing – original draft, Writing – review & editing.
